# Semaphorin 3 C drives epithelial-to-mesenchymal transition, invasiveness, and stem-like characteristics in prostate cells

**DOI:** 10.1038/s41598-017-11914-6

**Published:** 2017-09-13

**Authors:** Kevin J. Tam, Daniel H. F. Hui, Wilson W. Lee, Mingshu Dong, Tabitha Tombe, Ivy Z. F. Jiao, Shahram Khosravi, Ario Takeuchi, James W. Peacock, Larissa Ivanova, Igor Moskalev, Martin E. Gleave, Ralph Buttyan, Michael E. Cox, Christopher J. Ong

**Affiliations:** 1 Vancouver Prostate Centre, Vancouver General Hospital, 2660 Oak Street, Vancouver, BC V6H 3Z6 Canada; 2 Department of Urologic Sciences, University of British Columbia, Level 6, 2775 Laurel Street, Vancouver, BC V5Z 1M9 Canada

## Abstract

Prostate cancer (PCa) is among the most commonly-occurring cancers worldwide and a leader in cancer-related deaths. Local non-invasive PCa is highly treatable but limited treatment options exist for those with locally-advanced and metastatic forms of the disease underscoring the need to identify mechanisms mediating PCa progression. The semaphorins are a large grouping of membrane-associated or secreted signalling proteins whose normal roles reside in embryogenesis and neuronal development. In this context, semaphorins help establish chemotactic gradients and direct cell movement. Various semaphorin family members have been found to be up- and down-regulated in a number of cancers. One family member, Semaphorin 3 C (SEMA3C), has been implicated in prostate, breast, ovarian, gastric, lung, and pancreatic cancer as well as glioblastoma. Given SEMA3C’s roles in development and its augmented expression in PCa, we hypothesized that SEMA3C promotes epithelial-to-mesenchymal transition (EMT) and stem-like phenotypes in prostate cells. In the present study we show that ectopic expression of SEMA3C in RWPE-1 promotes the upregulation of EMT and stem markers, heightened sphere-formation, and cell plasticity. In addition, we show that SEMA3C promotes migration and invasion *in vitro* and cell dissemination *in vivo*.

## Introduction

Prostate cancer (PCa) is among the most commonly-occurring cancers worldwide and a leader in cancer-related deaths. Localized low grade PCa can be treated by surgical resection and radiation therapy and is typically met with favourable outcome. By contrast, locally-advanced and metastatic forms of PCa require more aggressive treatments that include combinations of hormone and chemo-based therapies as well as antiandrogens and inhibitors of steroidogenesis. However, these treatments are not curative. Despite initial tumour response in most cases to androgen-deprivation therapy, antiandrogens, inhibitors of steroidogenesis, and chemotherapies, relapse invariably occurs. The precise events leading to stages of treatment-resistance and disease progression are the subject of intensive investigation but one line of thought posits that a sub-population of ‘tumour-initiating cells’ within the larger tumour population are responsible for subverting standard radiation and chemotherapies and subsequently seeding relapse and metastasis^1–6^. Strategies targeting the tumour-initiating population of cells therefore hold potential utility in the clinic, particularly for those in advanced treatment-refractory stages of the disease.

Epithelial cells grow in juxtapose formation, exhibit cobblestone morphology, and display tight cell-cell junctions which function in maintaining tissue integrity and impermeability. By contrast, cells of the mesenchyme grow more loosely, show high motility, and exhibit minimal cell-cell junctions and cell polarity. Epithelial-to-mesenchymal transition (EMT) is a cellular process executed during embryogenesis and morphogenesis and is characterized by the transition of cells from an epithelial phenotype to a mesenchymal phenotype. There is strong support for the notion that cells commandeer this program on their way to becoming cancerous^7^. Doing so would grant the cancer cells with numerous characteristics necessary for the metastatic process including the ability to degrade basement membranes and extracellular matrix, migrate away from the primary tumour, survive dissemination to distant anatomical sites by way of lymphatic or circulatory systems, and extravasate & colonize foreign microenvironments. During EMT, shifts in the expression of cell-surface proteins, cytoskeletal proteins, and EMT-driving transcription factors can be observed, which collectively, confer the mesenchymal phenotype (typified by invasive and migratory behavior)^7^. Biomarkers and phenotypes of mesenchymal cells can be seen in tumour tissue. Interestingly, cells that have undergone an EMT share strikingly similarities to cancer stem cells and recent reports indicate a close relationship between the two programs^8–13^.

Evidence for a tumour-initiating cell or ‘cancer stem cell’ (CSC) was first provided by Bonnet and Dick in acute myeloid leukemia where it was shown that the CD34 + /CD38- subset of cells exhibits high tumour-initiating efficiency in mice^1^. Since Bonnet and Dick first demonstrated the existence of a CSC, others have shown similar sub-populations of cells with high tumour-initiating capacity in other solid cancers^2, 14–16^. One poorly understood aspect of this theory is the cell of origin that gives rise to this population. One possibility is that normal stem cells undergo neoplastic transformation followed by population expansion. Another possibility is that differentiated cells acquire stem-like phenotypes through a process of dedifferentiation where inadvertent reactivation of dormant stem programs, normally reserved for embryogenesis, confer the cell with newfound stem-like phenotypes. In any event, the so-called cancer stem cells share many qualities with normal stem cells such as potency and self-renewal and their existence is supported by tumour heterogeneity, poor differentiation of tumour tissue upon histological examination, and an overlap in gene expression profiles^17^. Another parallel can be drawn in the fact that the reconstitution of an organ by normal stem cells somewhat resembles tumour formation by a cancer stem cell in the heterogeneity displayed by both end products and the overall regenerative and prolific nature of the processes.

The semaphorins are membrane-associated or secreted chemotactic proteins with critical roles in development. The semaphorin family of signalling proteins were originally described for their roles in axon guidance in the developing nervous system^18, 19^ where they form molecular gradients and coordinate directional growth of axons toward or away from the areas they delineate^20^. Semaphorins are separated into 8 classes: classes 1, 2, and 5 are found in invertebrates, 3 through 7 are found in vertebrates, and the eighth class, designated V, is found in viruses. All semaphorins contain a characteristic N-terminal 500 amino acid sema domain which folds into a seven-bladed β-propeller^21^. The sema domain is involved in protein-protein interactions with its receptors, the plexins (PLXNs) and neuropilins (NRPs)^22–25^. PLXNs have large cytoplasmic domains and are generally thought to be the signal-transducing molecule for the semaphorins^22, 26^. Semaphorins act through autocrine, paracrine, and juxtacrine signalling and have been implicated in a broad range of biological functions ranging from tissue morphogenesis to immunity^27, 28^; however, their association in malignancies is becoming increasingly evident^29^.

SEMA3C was initially discovered for its roles in neurogenesis and cardiac development^30, 31^ and plays crucial roles in cell migration in the neural crest^32^. SEMA3C has also been implicated in prostate, breast, ovarian, gastric, lung, and pancreatic cancer, as well as glioblastoma^29^. The receptors to SEMA3C include NRP1, NRP2, PLXNB1, and PLXND1^33–35^. PLXNB1 has been shown to activate c-Met while PLXND1 has been shown to activate ErbB2 and EGFR signalling and promote invasiveness and metastatic spread *in vivo* ^36, 37^. Neuropilins, on the other hand, have been shown to activate VEGFR signalling^38^. SEMA3C is upregulated in response to chemotherapy and radiation treatment^39^, promotes metastasis to the lung^40^, and promotes tumourigenicity of glioma cells^41, 42^. SEMA3C has also been shown to increase cell proliferation and migration, decrease apoptosis, and promote integrin signalling and VEGF secretion in endothelial cells^43^. SEMA3C was shown to drive migration of breast cancer cells^44^ and more recent studies have highlighted the importance and prognostic value of SEMA3C in pancreatic cancer and PCa^45–48^. Specifically, SEMA3C predicts shorter time to biochemical recurrence in histopathological samples of low- and intermediate-risk PCa. Others have shown that SEMA3C decreases E-cadherin expression and increases invasiveness of the prostate cancer cell line PC-3 cells. Despite compelling evidence implicating SEMA3C in PCa, a complete understanding of its roles in PCa etiology remains to be clearly defined. Our work sets out to identify the details through which SEMA3C drives PCa progression. SEMA3C’s amplification in PCa combined with its known roles in development and morphogenesis led us to hypothesize that SEMA3C promotes PCa through activation of EMT and stem programs. Our results demonstrate that SEMA3C overexpression in RWPE-1 prostate cells causes an upregulation of EMT and stem markers which is accompanied by invasiveness and stem-like phenotypes both *in vitro* and *in vivo*.

## Results

### Generation of RWPE-1 cells stably overexpressing SEMA3C

**Figure 1:**
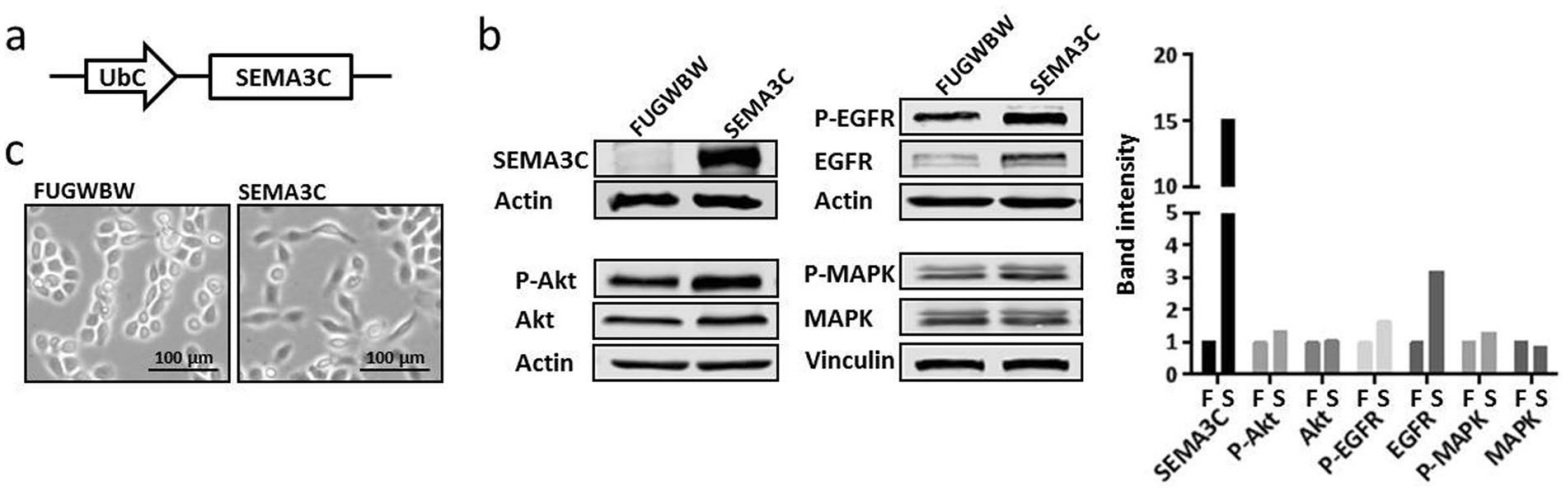
Generation of RWPE-1 cells stably overexpressing SEMA3C. SEMA3C was cloned under the control of a human Ubiquitin C promoter in a modified FUGW lentiviral vector designated FUGWBW using Gateway technology (**a**) (Invitrogen). Immortalized normal prostate epithelia RWPE-1 cells were transduced with virus made from either a SEMA3C overexpression construct to achieve constitutive expression (SEMA3C) or empty parental vector to serve as a control (FUGWBW). Overexpression of SEMA3C was confirmed by Western blot analysis of cell lysate where actin served as loading control (**b**). Phospho- and total levels of Akt, EGFR, and MAPK were also examined where actin or vinculin served as loading control. Western blot band intensity was quantitated by densitometry (**b**, right); ‘F’ = FUGWBW, ‘S’ = SEMA3C. Control cells showed cobblestone morphology which is characteristic of epithelia while SEMA3C-overexpressing cells showed cobblestone and spindle-like morphologies (**c**).

We chose the non-transformed prostate epithelial cell line, RWPE-1, for our studies due to their low intrinsic Semaphorin 3 C (SEMA3C) levels and strong epithelial phenotypes. *SEMA3C* was cloned downstream of the human Ubiquitin C (UBC) promoter (Fig. 1a) in a modified FUGW lentiviral vector (FUGWBW). RWPE-1 cells were transduced with lentivirus to achieve stable overexpression of SEMA3C. These cells (referred to as ‘SEMA3C’) produced substantially more SEMA3C than parental vector-transduced cells (referred to as ‘FUGWBW’) which was confirmed by Western blot analysis of their whole cell extract (Fig. 1b). We examined potential activation of growth-promoting signalling pathways and found that SEMA3C overexpression led to modest increases in phospho-Akt and phospho-EGFR. Interestingly, total EGFR levels dramatically increased. SEMA3C overexpression did not alter levels of phospho-MAPK. Band intensity was quantitated by densitometry (Fig. 1b, right). Overexpression of SEMA3C in RWPE-1 was also associated with morphological changes as captured by bright-field microscopy (Fig. 1c). RWPE-1-FUGWBW cells exhibited cobblestone morphology typical of epithelial cells, however, the RWPE-1-SEMA3C cell population contained both cobblestone-shaped cells and a population of cells with spindle-like morphology reminiscent of mesenchymal cells. The changes in morphology are consistent in the semaphorins’ known roles in cellular morphology and cytoskeletal rearrangements^18–20, 33, 49^. Morphological changes became increasingly evident (with respect to the proportion of cells displaying this characteristic) as a function of passage number suggesting enrichment of a population rather than a uniform transition by the entire population or transdifferentiation.

### Overexpression of SEMA3C causes an upregulation of EMT markers

**Figure 2:**
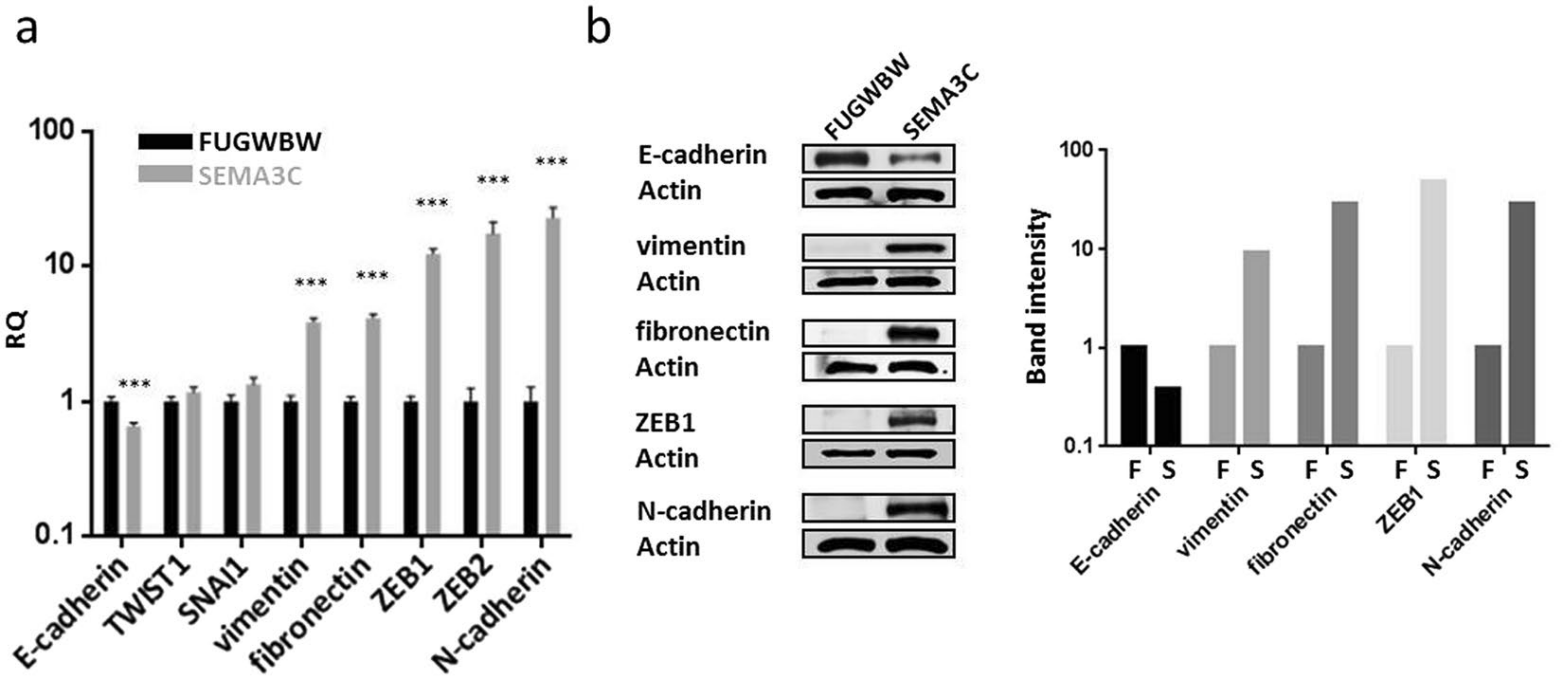
Overexpression of SEMA3C causes an upregulation of EMT markers. Expression levels of a panel of EMT markers were compared between SEMA3C-overexpressing RWPE-1 cells (RWPE-1-SEMA3C) and control cells (RWPE-1-FUGWBW). Significant upregulation of N-cadherin, ZEB2, ZEB1, fibronectin, vimentin, and downregulation of E-cadherin was observed in RWPE-1-SEMA3C compared to control RWPE-1-FUGWBW as shown by qPCR. (**a**) Data represent mean, ± SD; ***p < 0.001 comparing RWPE-1-SEMA3C to RWPE-1-FUGWBW cells. These findings were verified by Western blot analysis; (**b**) Western blot band intensity was quantitated by densitometry (b, right); ‘F’ = FUGWBW, ‘S’ = SEMA3C.

The inappropriate execution of EMT has been proposed to be a root cause of metastasis. Trademarks of EMT include diminished cell-to-cell contacts, loss of cellular polarity, and increased cell motility. The loss of cobblestone morphology and decreased cell-cell contacts by SEMA3C-overexpressing RWPE-1 cells are indicative of EMT. If an EMT were occurring, it would also be reflected in changes in the expression of EMT-associated genes. To examine this possibility, we compared the expression of a panel of EMT markers in RWPE-1-FUGWBW and RWPE-1-SEMA3C cells. Quantitative polymerase chain reaction (qPCR) indicated that overexpression of SEMA3C caused an upregulation of N-cadherin, ZEB2, ZEB1, fibronectin, and vimentin, and a down-regulation of E-cadherin (Fig. 2a). Other EMT-associated transcription factors such as TWIST1 and SNAI1 did not drastically change. The changes in expression of these genes were confirmed at the protein level by Western blot analysis (Fig. 2b). Band intensity was quantitated by densitometry (Fig. 2b, right). We were unable to confirm ZEB2 levels by Western blot due to the lack of an effective commercially-available antibody. These results show that overexpression of SEMA3C promotes epithelial-to-mesenchymal transition.

### SEMA3C increases migration and invasion *in vitro*

**Figure 3:**
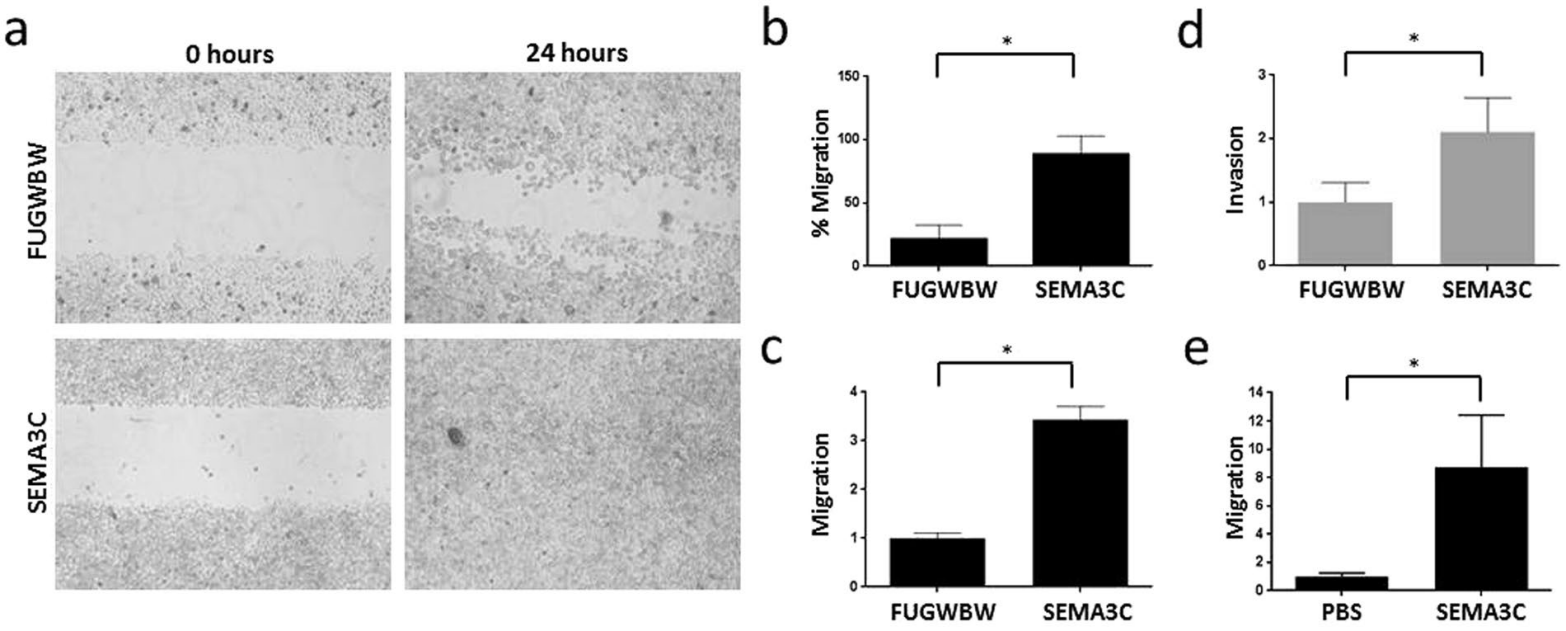
SEMA3C increases migration and invasion *in vitro*. The migration of RWPE-1-SEMA3C was compared to that of control cells by wound-healing assay. (**a**) % Migration in wound-healing assay was quantitated by the formula [(pixels at T_0h_) − (pixels at T_24h_)]/(pixels at T_0h_) × 100%. (**b**) In transwell migration assay, RWPE-1-SEMA3C cells were roughly 3.5 times more motile than control cells; (**c**) y-axis is fold increase in migration over control cells. In Matrigel Invasion assays, RWPE-1-SEMA3C cells were 2 times more invasive than control cells; (**d**) y-axis is fold increase in invasion over control cells. RWPE-1-FUGWBW cells migrated more strongly toward SEMA3C (1 μM) than to PBS; (**e**) y-axis is fold increase in migration over PBS. Data represent mean, ± SD; *p < 0.05 compared to control.

Mesenchymal cells exhibit motile and invasive behaviour which may trigger the onset of metastatic disease. As we found that RWPE-1-SEMA3C cells displayed a typical EMT gene expression signature, we next sought to determine if this was accompanied by phenotypic changes. In wound-healing assays, RWPE-1-SEMA3C cells were able to close the wound more rapidly than RWPE-1-FUGWBW cells (Fig. 3a). RWPE-1-FUGWBW cells closed the wound by ~20% after a 24 hour period whereas RWPE-1-SEMA3C cells closed the wound an average of 80% over three biological replicates in the same time frame (Fig. 3b). RWPE-1-SEMA3C also exhibited greater migration in transwell migration assays (Fig. 3c). To assess the invasiveness of RWPE-1-SEMA3C cells, we performed a Matrigel Invasion assay and observed that RWPE-1-SEMA3C cells were approximately two times more invasive than control cells (Fig. 3d). RWPE-1-FUGWBW cells migrated more strongly when placed in media containing recombinant SEMA3C than when placed in media containing vehicle, PBS (Fig. 3e). These results confirm SEMA3C’s known chemotactic roles and shed light on mechanistic details underpinning SEMA3C-induced migration. RWPE-1-FUGWBW cells similarly migrated more strongly when placed in SEMA3C-containing conditioned media than when placed in control conditioned media (Supplementary Fig. S1). Collectively this demonstrates that overexpression of SEMA3C in RWPE-1 cells promotes EMT at the molecular and phenotypic level.

### SEMA3C promotes cell dissemination *in vivo*

**Figure 4:**
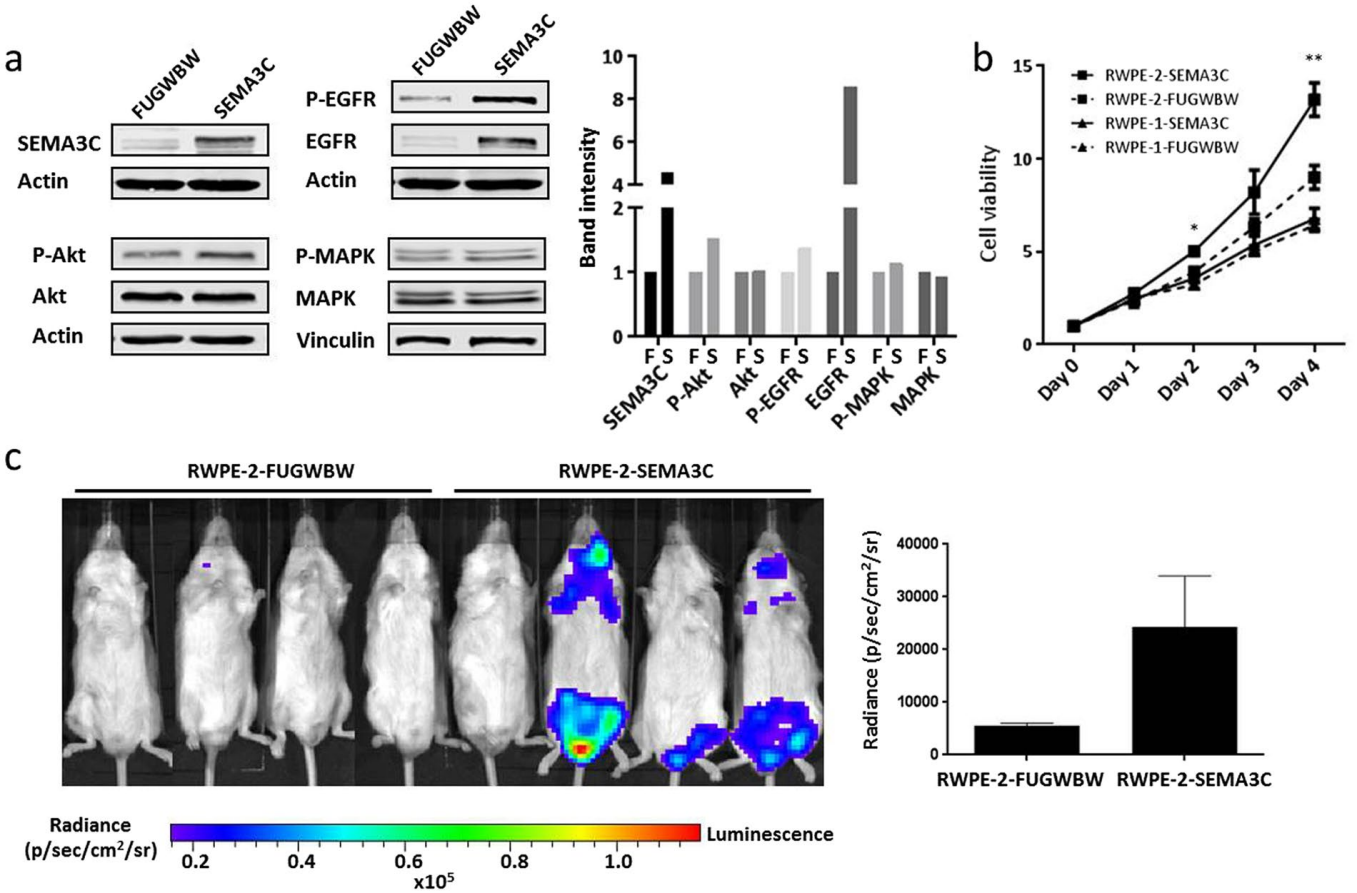
SEMA3C promotes cell dissemination *in vivo*. RWPE-2 cells were made to stably overexpress SEMA3C and firefly luciferase by lentiviral transduction to generate RWPE-2-FUGWBW and RWPE-2-SEMA3C. SEMA3C overexpression and phosphorylated and total levels of Akt, EGFR, and MAPK were examined by Western blot analysis. (**a**) Western blot band intensity was quantitated by densitometry (**a**, right); ‘F’ = FUGWBW, ‘S’ = SEMA3C. Cell viability of RWPE-1-FUGWBW, RWPE-1-SEMA3C, RWPE-2-FUGWBW, and RWPE-2-SEMA3C was compared; (**b**) data represent mean viability over day 1 ± SD; *p < 0.05, **p < 0.01 where significance is measured between RWPE-2-FUGWBW and RWPE-2-SEMA3C. (**c**) IVIS measurements of NOD scid gamma mice xenografted with RWPE-2-FUGWBW and RWPE-2-SEMA3C by ultrasound-guided intracardiac injection; measurements were taken at 7 weeks post-inoculation. Data represents mean radiance ± SEM; *p = *0.09995.

To determine if the invasiveness we observed in *in vitro* studies translated to more aggressive tumour dynamics *in vivo*, we compared the metastatic potential of RWPE-2 cells overexpressing SEMA3C to mock transduced cells. To overcome the inherently low tumour-initiating capabilities of RWPE-1 cells, we utilized RWPE-2 cells for these studies. RWPE-2 are derived from the RWPE-1 and are transformed by virtue of infection by the Kirsten murine sarcoma virus and Ki-Ras^50^. RWPE-2 cells are tumourigenic *in vivo* whereas RWPE-1 cells are not. When SEMA3C was overexpressed in RWPE-2, levels of phospho-Akt, phospho-EGFR, and total EGFR increased whereas levels of phospho-MAPK did not (Fig. 4a); these findings mirror those seen in RWPE-1 (Fig. 1b). Band intensity was quantitated by densitometry (Fig. 4a, right). In cell viability assays, RWPE-2-SEMA3C displayed the most aggressive growth kinetics followed by RWPE-2-FUGWBW and finally RWPE-1-FUGWBW and RWPE-1-SEMA3C which grew at approximately the same rate (Fig. 4b). Given that SEMA3C overexpression promoted cell growth on the RWPE-2 background and not on the RWPE-1 background combined with the knowledge that the Ki-Ras oncogene is a feature unique to the RWPE-2 cells, this would suggest that SEMA3C may cooperate with Ras to accelerate growth during oncogenesis. For *in vivo* studies, cells were engineered to also express luciferase. Cells were introduced by ultrasound-guided intracardiac injection of NOD scid gamma mice and monitored for tumour formation by *in vivo* imaging system (IVIS). Seven weeks after injection, three of four mice injected with RWPE-2-SEMA3C cells displayed tumours in the head and groin region by IVIS while zero of four mice injected with RWPE-2-FUGWBW formed tumours (Fig. 4c) nor did tumours form in mice xenografted with recombinant RWPE-1 cells (Supplementary Fig. S2). Quantitation of luminescence in xenografted mice is shown (Fig. 4c, right). These results would suggest that SEMA3C promotes cell dissemination *in vivo*.

### Overexpression of SEMA3C promotes stem-like characteristics

**Figure 5:**
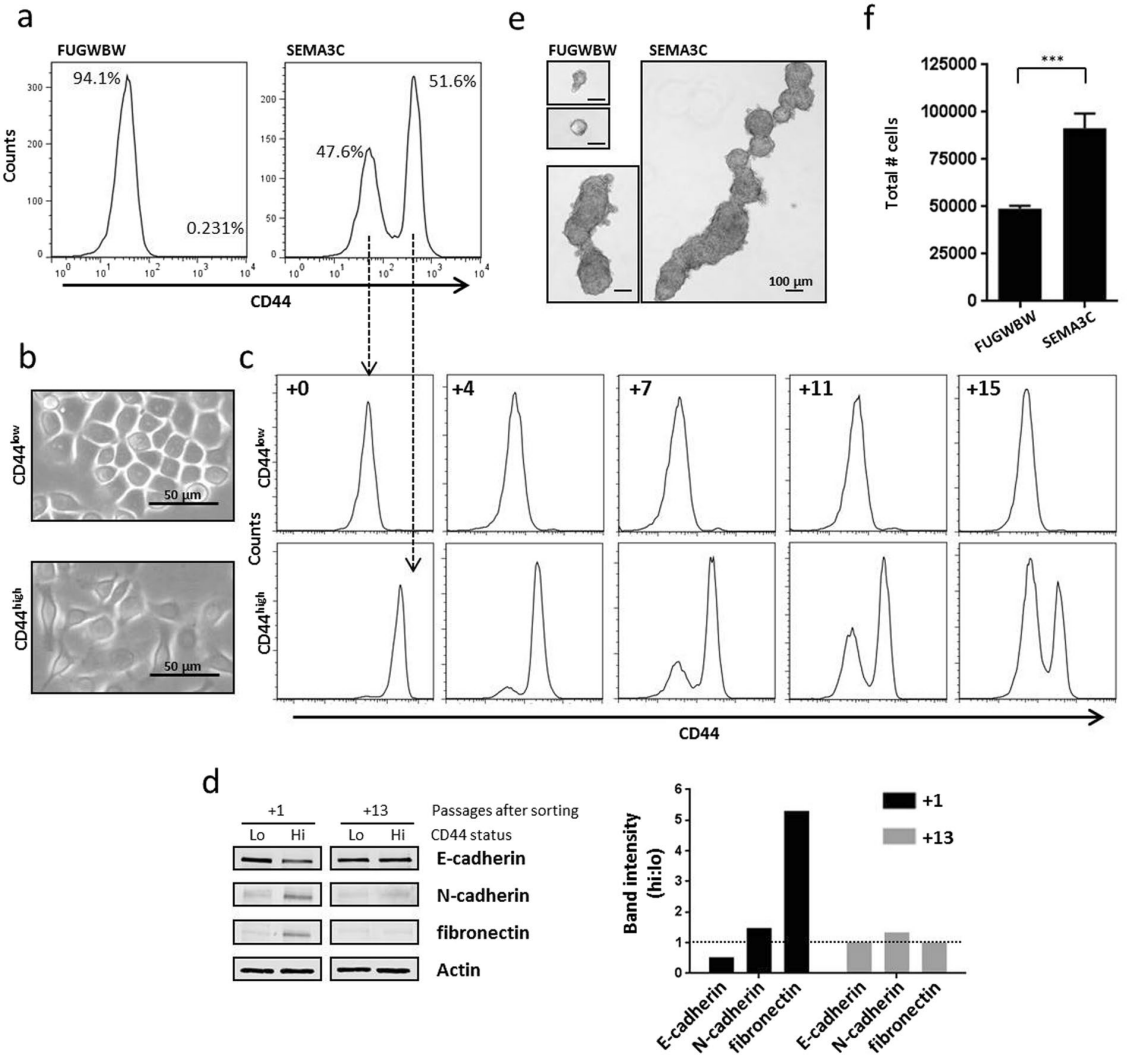
Overexpression of SEMA3C promotes stem-like characteristics. RWPE-1-FUGWBW cells expressed detectable levels of the PCa stem cell marker CD44 as shown by flow cytometry. However, RWPE-1-SEMA3C contained two distinguishable CD44 populations, a CD44^low^ population and a CD44^high^ population. (**a**) The two CD44 cell populations within the RWPE-1-SEMA3C cell population were sorted on CD44 status; CD44^low^ cells were cobblestone in morphology (**b**) and remained CD44-low (**c**) whereas CD44^high^ cells were spindle-shaped (**b**) and reconstituted the CD44^low^ population. (**c**) Numbers in the top left corner of FACS plots refer to the number of passages following cell sorting. Levels of E-cadherin, N-cadherin, and fibronectin in CD44^low^ and CD44^high^ cells were examined by Western blot at one (+1) and thirteen (+13) passages following sorting. (**d**) Based on densitometry, ratios of E-cadherin, N-cadherin, and fibronectin between CD44^high^ and CD44^low^ are presented graphically (**d**, right). Sphere-formation is used as an *in vitro* measure of stemness; while RWPE-1-FUGWBW cells were capable of forming modestly sized spheres which existed as solitary or aggregates of three or fewer spheres, RWPE-1-SEMA3C cells formed larger spheres which was likely the result of coalescence of many individual spheres. (**e**) To quantitate sphere-forming abilities, spheres were dissociated and constituent cells were counted by hemocytometer. RWPE-1-SEMA3C cells formed spheres roughly two times more efficiently than control cells when evaluated in this way; (**f**) results are representative of three independent experiments. Data represent mean, ± SD; ***p < 0.001 compared to RWPE-1-FUGWBW control.

It has been proposed that a stem-like population of cells is responsible for causing tumour relapse and subsequent metastatic disease. The so-called cancer stem cell would be uniquely capable of self-renewal and would exhibit potency and anchorage-independent growth. Given SEMA3C’s known roles in development and morphogenesis we hypothesized that SEMA3C drives PCa progression by contributing aspects of the cancer stem cell phenotype that has been operationally defined by others^8–13, 51^. CD44 is a cell-surface glycoprotein initially described for its expression on leukocytes and its affinity for extracellular matrix proteins such as hyaluronic acid. CD44’s cellular functions relate to migration, cell proliferation, and cell survival^52^. CD44 has also been extensively used as a marker for cancer stem cells^2, 14, 53^. To test whether RWPE-1-SEMA3C cells expressed elevated CD44, we used flow cytometry to examine CD44 status. FACS analysis showed that control cells were CD44^low^ while RWPE-1-SEMA3C harboured both CD44^low^ and CD44^high^ populations (Fig. 5a). To evaluate the plasticity of the CD44^low^ and CD44^high^ cell populations within the RWPE-1-SEMA3C cells, cells were sorted based on their CD44 status. CD44^low^ cells were cobblestone in morphology (Fig. 5b) and remained low in CD44 expression upon passaging (Fig. 5c) whereas the CD44^high^ cells were spindle-shaped (Fig. 5b) and reconstituted the CD44^low^ population over successive passages (Fig. 5c). At one passage after sorting, CD44^high^ cells expressed lower E-cadherin, higher N-cadherin, and higher vimentin than CD44^low^ cells. At thirteen passages after sorting, the CD44^high^ cells displayed roughly the same amount of these proteins as the CD44^low^ cells (Fig. 5d). Densitometric ratios between the CD44^high^ and CD44^low^ cells are represented graphically (Fig. 5d, right). The two CD44 populations that arose from the CD44^high^-sorted cells were again sorted on CD44 status and similar results were obtained (Supplementary Fig. S3). Sphere-formation is an *in vitro* measure of stemness and exploits anchorage-independent cell growth; the ability of a gene to drive anchorage-independent growth is considered to be one of the defining characteristics of cellular oncogenesis. In the sphere-formation assay, we observed that RWPE-1-SEMA3C cells displayed superior sphere forming ability than the control cells with respect to sphere size (Fig. 5e) and number of constituent cells (Fig. 5f). Similarly, parental RWPE-1 cells grown as spheres exhibit higher SEMA3C expression than their adherent counterparts (Supplementary Fig. S4). Collectively this suggests that SEMA3C promotes the formation of a stem-like population of cells.

### Co-expression of CD44 and EMT markers on SEMA3C-overexpressing cells

**Figure 6:**
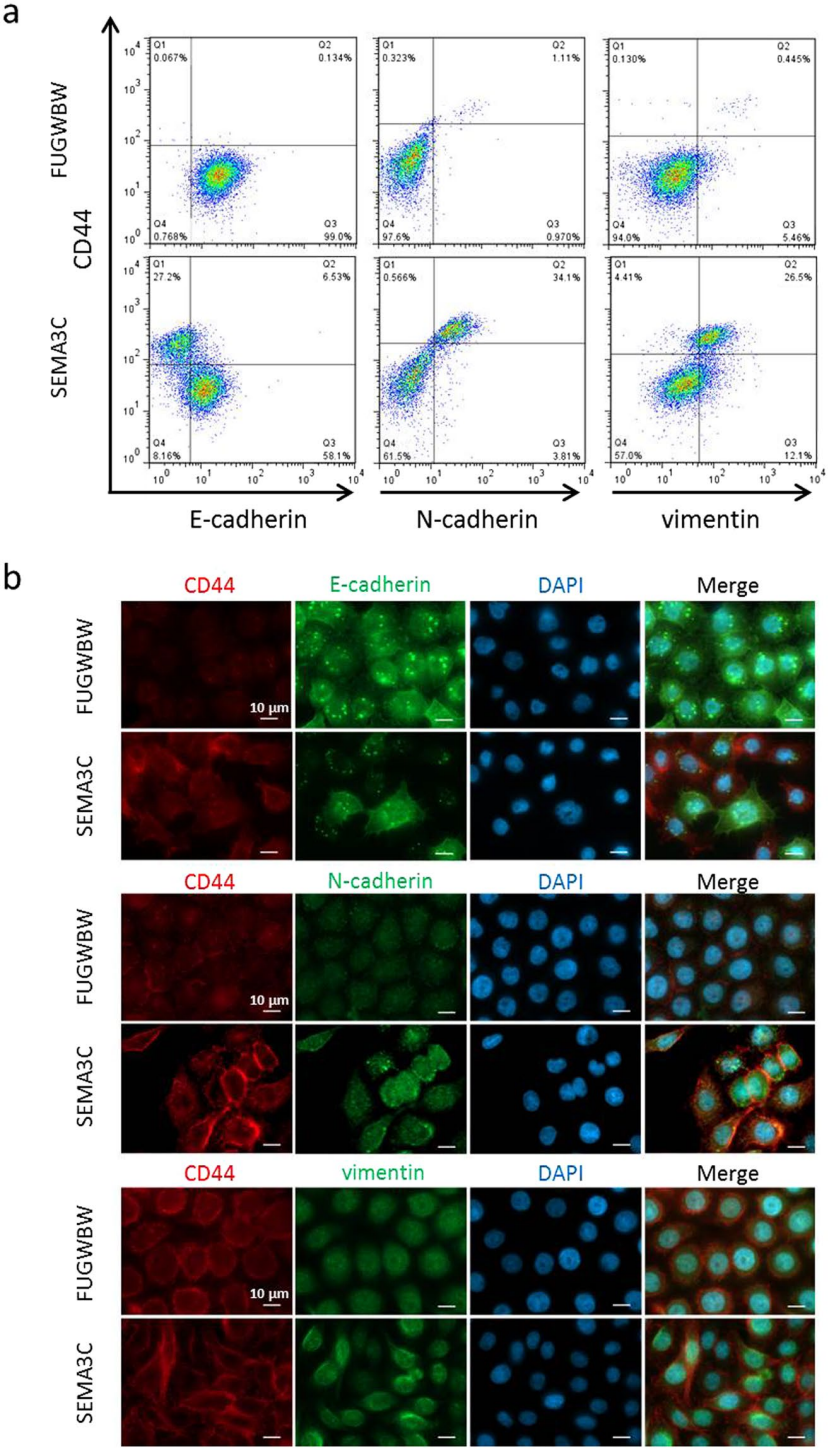
Co-expression of EMT and stem markers on SEMA3C-overexpressing cells. Co-expression of EMT markers and CD44 was determined by co-staining studies followed by flow cytometry. Within the RWPE-1-SEMA3C cell population, high CD44 expression was associated with low E-cadherin, high N-cadherin, and high vimentin expression while low CD44 expression was associated with high E-cadherin, low N-cadherin, and low vimentin expression. (**a**) Co-expression of mesenchymal and stem markers was confirmed by immunofluorescence microscopy where an inverse staining relationship existed between CD44 and E-cadherin and a positive staining relationship existed between CD44 and N-cadherin and between CD44 and vimentin (**b**).

Although classically considered separate processes, accumulating evidence would suggest that EMT and stemness are linked events^8–13^. We therefore sought to determine if RWPE-1-SEMA3C cells concurrently expressed EMT and stem markers. When we co-stained for CD44 and various EMT markers, we observed that cells inversely expressed CD44 and E-cadherin and co-expressed CD44 and N-cadherin and also CD44 and vimentin which was shown by flow cytometry (Fig. 6a). Co-expression studies were confirmed by immunofluorescence which similarly showed an inverse staining relationship between CD44 and E-cadherin and a positive relationship between CD44 and N-cadherin and between CD44 and vimentin within the RWPE-1-SEMA3C population (Fig. 6b). These findings support the notion that a common pool of cells possess both stem and EMT characteristics and are in agreement with findings by others.

## Discussion

We have demonstrated that ectopic expression of SEMA3C leads to the development of EMT and stem-like characteristics in the RWPE-1 cell line and in doing so illuminate potential roles of SEMA3C in PCa carcinogenesis. Overexpression of SEMA3C triggered changes in cell morphology, expression of EMT and stem markers, and acquisition of invasive and stem-like phenotypes. The notion that class 3 semaphorins can drive EMT is not altogether novel^45, 54^ nor are the links between SEMA3C and the stem phenotype^42, 55^. It is possible that vestiges of SEMA3C’s embryonic programs are unwittingly unleashed to drive PCa progression. Due to their roles in cell motility and cytoskeletal rearrangement, it is conceivable that SEMA3C could contribute to the EMT process. As the PCa stem cell is described to be a CD133, α2β1, and CD44 positive cell, future studies will need to examine whether SEMA3C is capable of also upregulating expression of CD133 and α2β1. SEMA3C-mediated upregulation of CD44 may depict a partial execution of the stem program with still other events being required to complete the transition. Normal and cancer stem cells can also be functionally characterized by their expression of aldehyde dehydrogenase (ALDH1), an enzyme that is enriched for in progenitor populations. ALDH1 levels correlate with poor prognosis in breast cancer and is thought to contribute to treatment resistance^56^. Consequently, it should be investigated whether SEMA3C overexpression corresponds to increased ALDH1 levels or activity.

Although the causative role of both EMT and stemness in cancer progression remains highly disputed, our findings may still hold clinical utility due to the fact that SEMA3C predicts biochemical recurrence in PCa and enhances invasiveness of prostate cancer cells^45, 47^. Outside of PCa, SEMA3C has been shown to cause metastasis to the lung^40^ and support growth and tumourigenicity of glioma stem cells^42^. The semaphorin family of proteins are implicated in virtually every Hallmark of Cancer^29^ and SEMA3C specifically is frequently associated with cancer growth^57^. SEMA3C is an attractive target in this regard because SEMA3C’s roles lie primarily in development and their functions in adults are diminished^33^. Furthermore, as the biologically active form of SEMA3C is secreted, the fraction of SEMA3C that is accessible for targeting by pharmacological agents is high.

As lentiviral transduction involves random integration of viral DNA into the genome, it is possible that catastrophic integration events that inactivated tumour suppressors such as *TP53* or *RB1* or activation of oncogenes are what led to disruption of cell homeostasis and transformation. To rule out these chance integration events as the cause for our results, lentiviral transduction of RWPE-1 with SEMA3C-overexpression and control vectors was repeated a second time with similar results being observed (Supplementary Fig. S5). Limitations to our study include the fact that these findings have been demonstrated in only the RWPE-1 cell line. Thus, studies in more diverse cell types will prove useful in discerning context-specific versus universal principles of semaphorin biology. Although we have observed that SEMA3C induces upregulation of CD44 in multiple cell lines (Supplementary Fig. S6), recent reports have shown that SEMA3C inhibits metastasis of cancer cells through inhibiting angiogenesis and lymphangiogenesis^58, 59^. Discrepant SEMA3C function between the RWPE-1 results presented here and those shown elsewhere may simply relate to the particular set of cognate receptors, proteolytic enzymes, or modified forms of SEMA3C present in the systems examined. The biological activities of semaphorins are refined by proteolytic cleavage and post-translational modifications and are likely further fine-tuned by the repertoire of NRP and PLXN receptors present on the recipient cell. Thus inconsistent or opposing semaphorin actions across different cell types may simply reflect different combinations, relative proportions, or absolute quantities of proteolytic enzymes, glycosyltransferases, PLXNs, and NRPs expressed by the cell types in question. Studies in more cell types and which correlate semaphorin activity with the receptors present will be useful, as will correlations between activity and semaphorin processing.

Increased levels of total EGFR in response to SEMA3C overexpression was repeatedly observed in our studies. This phenomenon warrants further investigation. SEMA3C-induced upregulation of EGFR could have profound implications in cancer development and could underpin our findings and those of studies involving semaphorins elsewhere. Additional signalling studies involving receptor tyrosine kinases like ErbB2 and c-Met and other substrates such as Src, Shc, and PI3K will shed light on mechanistic details surrounding SEMA3C activity. We predict these studies will show convergence of SEMA3C signalling networks with other well-characterized stem and EMT pathways. It is known that the semaphorins and their receptors, the plexins, can activate receptor tyrosine kinases such as c-Met, ErbB2, and VEGFR^36, 37, 43, 60, 61^. Additionally, semaphorin signalling can activate Akt, MAPK, Src, and PI3K signalling pathways^62, 63^ but the precise events that mediate SEMA3C-induced stem-like characteristics and EMT remain to be delineated. Such questions as whether or not SEMA3C signalling intersects any of the classical stem pathways (Wnt/β-catenin, hedgehog, notch, or TGFβ) also remains to be seen.

We have shown that ectopic expression of SEMA3C triggers the upregulation of stem and EMT markers in RWPE-1 cells and that this was accompanied by an increase in sphere forming ability, cell plasticity, motility, and invasiveness. We propose that SEMA3C-induced stem-like characteristics and EMT cooperate with other pathways, such as the androgen receptor axis, to drive PCa progression. While SEMA3C, EMT, and stemness have been separately discussed as contributing factors in PCa, no link has been made between these three topics in driving cancer progression and therefore these findings may be of clinical significance.

## Methods

### Cell lines and plasmids

SEMA3C (accession # NM_006379) or luciferase was cloned under the control of a human Ubiquitin C promoter in a modified lentiviral expression vector using Gateway Technology as described previously^64^. Cells were transduced with lentivirus generated from this plasmid as described previously^64^. RWPE-1 (ATCC, CRL-11609) and RWPE-2 (ATCC, CRL-11610) were cultured in KSFM (Invitrogen, 17005-042) supplemented with bovine pituitary extract to 0.05 mg/ml and 5 ng/ml human recombinant epidermal growth factor and maintained under Blasticidin S selection at 2 µg/ml. BPH-1 (kindly provided by Dr. S. Hayward, Vanderbilt University) and MDA-MB-468 (ATCC, HTB-132) were cultured in 10% FBS, DMEM and maintained under Blasticidin S selection as above. MCF 10A (ATCC, CRL-10317) were cultured in MEBM media + supplements supplied with Lonza CC-3150 and cholera toxin (SIGMA-ALDRICH, C8052) to 100 ng/ml and maintained under Blasticidin S selection as above.

### Western blot

Conditioned media or whole cell extracts were run on 10% acrylamide gels and transferred onto nitrocellulose membrane; lysates were prepared in 50 mM Tris-HCl, 150 mM NaCl, 1% NP40, 10 mM NaF, 10% glycerol, supplemented with protease inhibitor cocktail (Roche, 04693116001); protein concentration were determined using a BCA method (Thermo Scientific, 23228) and 50 µg of total protein was analyzed. Western blots were visualized using radiography film, a Syngene Dyversity, or a LI-COR Odyssey system. Antibodies for Western blot analysis are described in Supplementary Fig. S7. Actin and vinculin served as loading controls. Densitometry was calculated using GeneTools from Syngene version 4.03.05.0 normalizing to loading control.

### Quantitative polymerase chain reaction

Expression of EMT markers was assessed by qPCR. Total RNA was extracted using TRIzol (Invitrogen, 15596018). 2 µg of RNA was reverse-transcribed using random hexamers (Roche, R15504) and Superscript II (Invitrogen, 18064-014). qPCR was carried out using a ΔΔCt method on an AB ViiA7 real-time PCR machine; reactions were prepared using Platinum SYBR Green (Invitrogen, 11744-500) and GAPDH served as an endogenous control. See Supplementary Fig. S8 for primer sequences.

### Microscopy

Slides were fixed in 4% paraformaldehyde, PBS for 15 min., rinsed three times with PBS, blocked in 2% BSA, 0.2% Triton X-100, PBS for 30 min., stained with primary antibody diluted at 1/100-1/200 in 1% BSA, 0.2% Triton X-100, PBS for 2 hours or overnight at 4 degrees Celsius, rinsed three times with 0.05% Tween-20, PBS, incubated with secondary antibody diluted at 1/1,000 in 1% BSA, 0.2% Triton X-100, PBS for 1 hour, rinsed three times with 0.05% Tween-20, PBS, and finally fixed and DAPI-stained using ProLong Gold (Life Technologies, P36935). For double staining experiments, staining for targets occurred sequentially. Bright-field and immunofluorescent images were captured on a Zeiss AxioObserver.Z1 using ZEN Light Blue software. Antibodies for immunofluorescence are described in Supplementary Fig. S7.

### Migration and invasion assay

Cell migration was measured by wound-healing (‘scratch’) assay. Cells were seeded in 6-well plates and grown to confluency. Cells were pre-treated with Mitomycin C at 15 µg/ml for thirty minutes to inhibit cell proliferation. Following treatment with Mitomycin C, cells were mechanically scratched, changed to fresh medium, and imaged for the first time point. Cells were imaged at a second time point twenty-four hours later. Percent migration in wound-healing assay was calculated using the formula: [(pixels at T_0h_)-(pixels at T_24h_)] / (pixels at T_0h_) × 100% across three biological replicates. Migration was also measured using a Boyden chamber transwell migration assay (Costar, 3422) as per manufacturer’s instruction. Two-hundred thousand cells were seeded per chamber. Migrated cells were quantitated by treatment with Calcein, AM (Life Technologies, C3099) and measured on a TECAN Infinite F500 plate reader using i-control1.7 software. Invasion was measured using a BD Matrigel Invasion Chamber approach (BD Biosciences, 354480) the setup for which paralleled migration assay. For chemotactic migration assays, two-hundred thousand RWPE-1-FUGWBW cells were placed in the upper chamber and allowed to migrate toward PBS as control or recombinant SEMA3C (1 μM) or conditioned media from RWPE-1-FUGWBW (FUGWBW CM) as control or conditioned media from RWPE-1-SEMA3C (SEMA3C CM) which was placed in the well beneath the chamber. Recombinant SEMA3C was purified from conditioned media of CHO-S cells stably transduced to overexpress SEMA3C using a HisTrap excel column (GE Healthcare). For conditioned media experiments, conditioned media was concentrated five times using centrifugal filters (Millipore, UFC901024).

### Flow cytometry

CD44 status was monitored using a BD Biosciences FACSCanto II. Cells were collected using trypsin and washed in FACS buffer (2% FBS, PBS) prior to antibody staining (30 min on ice). Antibodies can be found in Supplementary Fig. S7. Cells were washed three times with 0.5 ml FACS buffer after each antibody. Targets were probed sequentially for double staining. For intracellular staining, cells were fixed and permeabilized using Cytofix/Cytoperm (BD Biosciences, 51-2090KZ) and 0.2% Triton X-100, PBS, blocked and stained in 1% BSA, 0.05% Tween-20, PBS, and washed in 0.05% Tween-20, PBS. Stained and washed cells were brought up in 0.5 ml FACS buffer for running on flow cytometer. FSC: 250v, SSC: 375v, PerCP-Cy5 (CD44): 325v, FITC (E-cadherin, N-cadherin, vimentin): 400v. FlowJo Analysis software was used to analyze data. CD44^low^ and CD44^high^ cells were sorted using a FACSAria IIu.

### Sphere forming assay

For sphere (embryoid body)-forming assays ten-thousand cells were seeded in ultra-low attachment surface 6-well dishes (Corning, 3471) and cultured in MammoCult as per manufacturer’s instruction (STEMCELL Technologies, 04620). Spheres were allowed to develop for one week and then imaged on a Zeiss AxioObserver.Z1. To count constituent cells, spheres were dissociated by trypsin and counted by Trypan blue staining and hemocytometer.

### Proliferation Assay

Three thousand (RWPE-2) or six thousand (RWPE-1) cells were plated in black clear-bottom 96-well plates (Corning, 3904) in KSFM media containing supplements. Viability was measured using PrestoBlue Cell Viability Reagent (ThermoFisher Scientific, A-13261) as per manufacturer’s instruction and read on a TECAN Infinite F500 plate reader using i-control1.7 software.

### Animal studies

All animal experiments detailed within the manuscript were approved by the UBC Animal Care Committee, conforming to the mandatory guidelines of the Canadian Council on Animal Care. Experiments were carried out in accordance with UBC animal protocol number A15-0150. Assessment of *in vivo* tumourigenicity was carried out by ultrasound-guided (Vevo 770, VisualSonics) intracardiac injection of 5 × 10^5^ cells resuspended in PBS into NOD scid gamma (NSG) mice (The Jackson Laboratory, strain 005557). Tumour formation was monitored by intraperitoneal injection of mice with luciferin (Caliper Life Sciences, 119222) and bioluminescence readings on an *In Vivo* Imaging System (IVIS Lumina, PerkinElmer) using Living Image 4.2 software.

### Statistics

Statistical analysis was performed using the Student’s two-tailed t-test. Unless otherwise stated, data are represented as mean ± SD. Data presented are representative of three biological replicates.

### Data availability statement

The authors declare that any requested data or materials will be made available upon request.

## Electronic supplementary material

Supplementary Figures
