# Supplementary material for: Semaphorin 3 C drives epithelial-to-mesenchymal transition, invasiveness, and stem-like characteristics in prostate cells
Source: Sci Rep. 2017 Sep 13;7:11501. doi: 10.1038/s41598-017-11914-6 (PMC5597577; doi:10.1038/s41598-017-11914-6)
Supplement: Supplementary file 1 — Supplementary Figures [file 41598_2017_11914_MOESM1_ESM.pdf]

SUPPLEMENTARY DATA

Semaphorin 3C drives epithelial-to-mesenchymal transition, invasiveness, and stem-like characteristics in prostate cells

Kevin J. Tam[1,2], Daniel H. F. Hui[1], Wilson W. Lee[1], Mingshu Dong[1], Tabitha Tombe[1], Ivy Z. F. Jiao[1], Shahram Khosravi[1], Ario Takeuchi[1], James W. Peacock[1], Larissa Ivanova[1], Igor Moskalev[1], Martin E. Gleave[1,2], Ralph Buttyan[1,2], Michael E. Cox[1,2], and Christopher J. Ong[1,2]*

[1]Vancouver Prostate Centre, Vancouver General Hospital, 2660 Oak Street, Vancouver, BC, Canada, V6H 3Z6
[2]Department of Urologic Sciences, University of British Columbia, Level 6, 2775 Laurel Street, Vancouver, BC, Canada, V5Z 1M9

*Correspondence to: chris.ong@ubc.ca

Supplementary Figure S1. SEMA3C is a chemotactic agent

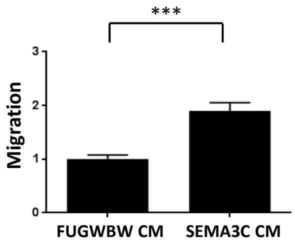

Supplementary Figure S1. SEMA3C is a chemotactic agent. RWPE-1-FUGWBW cells migrated more strongly toward conditioned media from SEMA3C-overexpressing RWPE-1-SEMA3C (SEMA3C CM) than to conditioned media from RWPE-1-FUGWBW (FUGWBW CM); y-axis is fold increase in migration over FUGWBW CM. Data represent mean, ± SD; *** $p < 0.001$.

Supplementary Figure S2. Intracardiac injection of NOD scid gamma mice with RWPE-1 stable cells

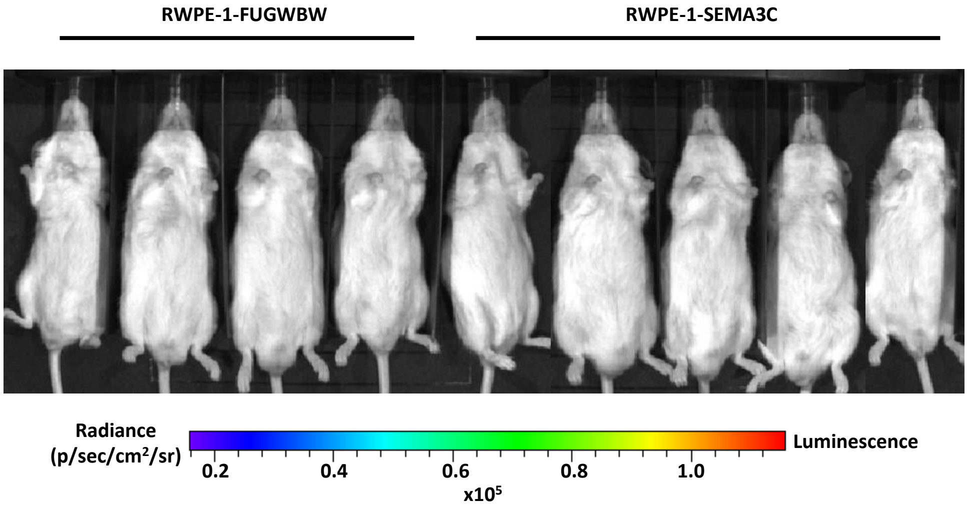

Supplementary Figure S2. Intracardiac injection of NOD scid gamma mice with RWPE-1 stable cells. $5x10^5$ luciferase-expressing RWPE-1-FUGWBW and RWPE-1-SEMA3C were injected by ultrasound-guided intracardiac injection and monitored for tumour formation by IVIS.

Supplementary Figure S3. RWPE-1-SEMA3C-CD44^{high} cells reconstitute the CD44^{low} population

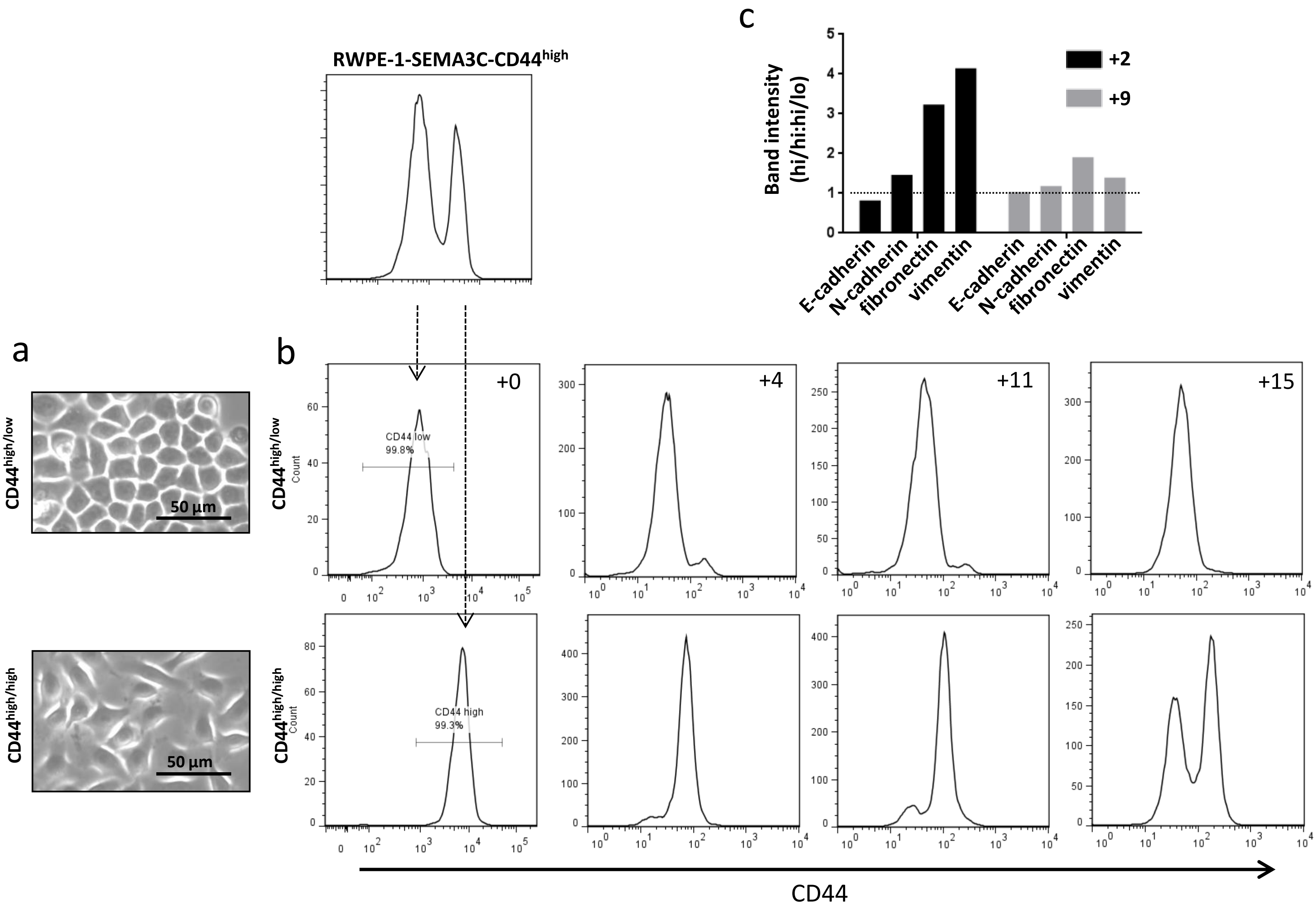

Supplementary Figure S3. RWPE-1-SEMA3C-CD44^{high} cells reconstitute the CD44^{low} population. CD44^{high} cells were re-sorted on CD44 status into 'CD44$^{high/low}$' and 'CD44$^{high/high}$' populations; CD44$^{high/low}$ cells were cobble stone in morphology (a) and remained CD44-low (b). CD44$^{high/high}$ cells were spindle-shaped (a) and reconstituted the CD44^{low} population (b). At two passages following sorting, CD44$^{high/high}$ cells were higher in N-cadherin, fibronectin, and vimentin expression and lower in E-cadherin expression than CD44$^{high/low}$ cells but the expression of these proteins became roughly equal by nine passages after sorting (c).

Supplementary Figure S4. SEMA3C is expressed at higher levels in RWPE-1 spheroids than in adherent RWPE-1

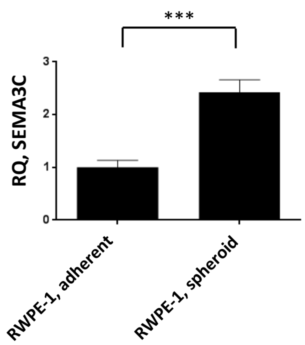

Supplementary Figure S4. SEMA3C is expressed at higher levels in RWPE-1 spheroids than in adherent RWPE-1. SEMA3C levels were compared between RWPE-1 cells plated under adherent conditions versus anchorage-independent conditions (as spheroids) by qPCR. Data represent mean, ± SD; *** $p < 0.001$.

Supplementary Figure S5. Repeat of RWPE-1 lentiviral transduction

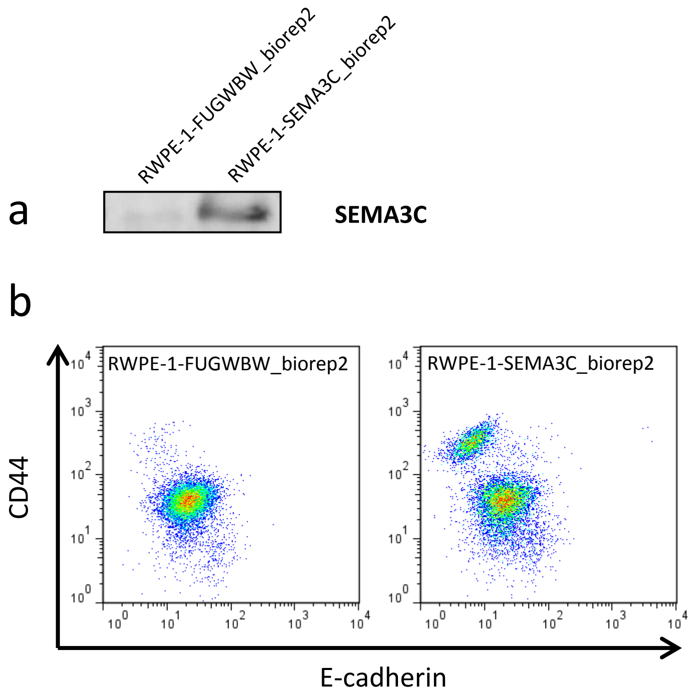

Supplementary Figure S5. Repeat of RWPE-1 lentiviral transduction. Lentiviral transduction of RWPE-1 cells was repeated. Overexpression of SEMA3C verified by Western blot of conditioned media (a). Upregulation of CD44 and its inverse staining relationship with E-cadherin was confirmed by FACS (b).

Supplementary Figure S6. Upregulation of CD44 in additional prostate and breast lines overexpressing SEMA3C

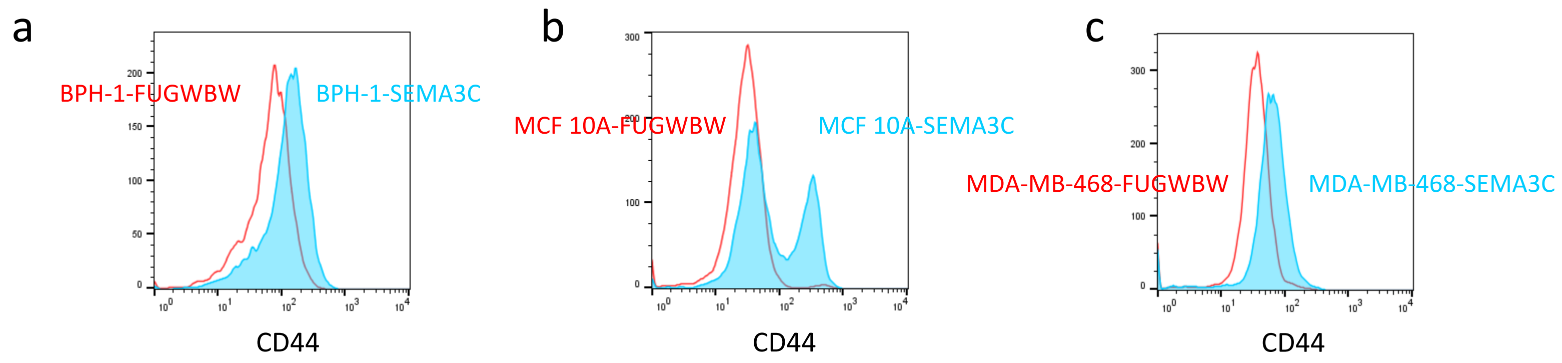

Supplementary Figure S6. Upregulation of CD44 in additional prostate and breast lines overexpressing SEMA3C. The CD44 status of an additional benign prostate line (BPH-1, a), a benign breast line (MCF 10A, b), and a breast cancer cell line (MDA-MB-468, c) stably overexpressing SEMA3C was documented by flow cytometry.

Supplementary Figure S7. Antibodies used

Antibodies:

	Ab	Company	Catalogue Number
**WESTERN BLOT**	Actin	SIGMA	A2066
	Vinculin	SIGMA	V4505
	SEMA3C (N-20)	Santa Cruz Biotechnology	sc-27796
	P-ERK	Cell Signaling Technology	4370S
	ERK	Cell Signaling Technology	4696S
	P-Akt	Cell Signaling Technology	4060S
	Akt	Invitrogen	44609G
	P-EGFR	Cell Signaling Technology	3777S
	EGFR	Santa Cruz Biotechnology	sc-377229
	E-cadherin	BD Transduction Laboratories	610181
	N-cadherin	BD Transduction Laboratories	610921
	vimentin	Cell Signaling Technology	3932S
	fibronectin	BD Transduction Laboratories	610077
	Zeb1	Cell Signaling Technology	3396S
	anti-goat HRP	Dako	P0160
	anti-rabbit HRP	Dako	P0448
	anti-mouse HRP	Dako	P0447
	Alexa Fluor 680 anti-rabbit IgG	Invitrogen	A21109
	Alexa Fluor 680 anti-mouse IgG	Invitrogen	A21058
**FLOW CYTOMETRY**	PE-Cy5 Isotype Control	eBioscience	15-4031-81
	CD44 PE-Cy5	eBioscience	15-0441-82
	E-cadherin (5H9)	Santa Cruz Biotechnology	sc-52327
	N-cadherin	BD Transduction Laboratories	610921
	vimentin	Cell Signaling Technology	3932S
	Alexa Fluor 488 anti-mouse IgG	Invitrogen	A11059
	Alexa Fluor 488 anti-rabbit IgG	Invitrogen	A11008
**IMMUNOFLUORESCENCE**			
	CD44 PE-Cy5	eBioscience	15-0441-82
	E-cadherin	BD Transduction Laboratories	610181
	N-cadherin	BD Transduction Laboratories	610921
	vimentin	Cell Signaling Technology	3932S
	Alexa Fluor 488 anti-mouse IgG	Invitrogen	A11059
	Alexa Fluor 488 anti-rabbit IgG	Invitrogen	A11008

Supplementary Figure S8. Primer sequences used

Primer sequences:

E-cadherin-F: 5'-GACAACAAGCCCGAATT-3'
E-cadherin-R: 5'-GGAAACTCTCTCGGTCCA-3'

N-cadherin-F: 5'-CGGGTAATCCTCCCAAATCA-3'
N-cadherin-R: 5'-CTTTATCCCGGCGTTTCATC-3'

vimentin-F: 5'-GAGAACTTTGCCGTTGAAGC-3'
vimentin-R: 5'-GCTTCCTGTAGGTGGCAATC-3'

fibronectin-F: 5'-CAGTGGGAGACCTCGAGAAG-3'
fibronectin-R: 5'-TCCCTCGGAACATCAGAAAC-3'

SNAl1-F: 5'-GCAAATACTGCAACAAGG-3'
SNAl1-R: 5'-GCACTGGTACTTCTTGACA -3'

TWIST1-F: 5'-GGAGTCCGCAGTCTTACGAG-3'
TWIST1-R: 5'-TCTGGAGGACCTGGTAGAGG -3'

ZEB1-F: 5'-TGCACTGAGTGTGGAAAAGC-3'
ZEB1-R: 5'-TGGTGATGCTGAAAGAGACG-3'

ZEB2-F: 5'-CGCTTGACATCACTGAAGGA-3'
ZEB2-R: 5'-CTTGCCACACTCTGTGCATT-3'

GAPDH-F: 5'-ATGACCCCTTCATTGACCTCA-3'
GAPDH-R: 5'-GAGATGATGACCCTTTTGGCT-3'
